# Effects of Prebiotics Inulin and Oat β-Glucan on Colonic Architecture and Hepatic Proteome in Mice with Circadian-Disruption-Aggravated Metabolic Dysfunction-Associated Steatohepatitis

**DOI:** 10.3390/nu17132245

**Published:** 2025-07-05

**Authors:** Nelson Kei, Kam Kuen Cheung, Ka Lee Ma, Tsz Kwan Yau, Susana Lauw, Xing Kang, Kiwi Wai Yan Sun, Yu Wang, Vincent Wai Sun Wong, Sunny Hei Wong, Peter Chi Keung Cheung

**Affiliations:** 1 Food and Nutritional Sciences Programme, School of Life Sciences, Faculty of Science, The Chinese University of Hong Kong, Hong Kong, China; jamescheung@link.cuhk.edu.hk (K.K.C.); kelly.kl.ma@cpce-polyu.edu.hk (K.L.M.); susana.lauw@link.cuhk.edu.hk (S.L.); 2 Food Research Centre, School of Life Sciences, Faculty of Science, The Chinese University of Hong Kong, Hong Kong, China; 3 Division of Science, Engineering and Health Studies, College of Professional and Continuing Education, The Hong Kong Polytechnic University, Hong Kong, China; 4 Cell and Molecular Biology Programme, School of Life Sciences, Faculty of Science, The Chinese University of Hong Kong, Hong Kong, China; tkyau@link.cuhk.edu.hk; 5 Lee Kong Chian School of Medicine, Nanyang Technological University, Singapore 308232, Singapore; jason.kangx@ntu.edu.sg (X.K.); sunny.wong@ntu.edu.sg (S.H.W.); 6 Department of Pharmacy, Queen Mary Hospital, Hospital Authority, Hong Kong, China; swy518@ha.org.hk; 7 Department of Pharmacology and Pharmacy, LKS Faculty of Medicine, The University of Hong Kong, Hong Kong, China; yuwanghk@hku.hk; 8 Department of Medicine and Therapeutics, Faculty of Medicine, The Chinese University of Hong Kong, Hong Kong, China; wongv@cuhk.edu.hk; 9 Department of Gastroenterology and Hepatology, Tan Tock Seng Hospital, Singapore 308433, Singapore

**Keywords:** prebiotic, inulin, β-glucan, metabolic dysfunction-associated steatohepatitis, circadian disruption, proteome

## Abstract

**Background:** Circadian disruption (CD) aggravates metabolic dysfunction-associated steatohepatitis (MASH), but supplementation with prebiotics inulin and oat β-glucan may mitigate its effects. However, their impact on colonic architecture and hepatic proteome remains unclear. **Objectives:** We aimed to investigate the effects of prebiotics inulin and oat β-glucan on colonic architecture and hepatic proteome in mice with CD-aggravated MASH. **Methods:** CD was induced by weekly reversal of the light–dark cycle to simulate shift work. Male C57BL/6J mice were subjected to non-shifted chow, non-shifted fructose, palmitate, cholesterol, and trans-fat (FPC) diet, shifted chow, or shifted FPC diet (SFPC) for 26 weeks. Prebiotics inulin and oat β-glucan supplementation were provided to the SFPC group in the final 10 weeks. Distal colon and serum samples were collected for histological examination and endotoxemia evaluation, respectively. Liver samples were collected for proteomic mass spectrometry analysis. **Results:** Mice with CD-aggravated MASH were found with colonic crypt loss and a distinct hepatic proteome structure compared to mice with non-CD MASH. Notably, inulin showed better effects than oat β-glucan in preserving colonic crypts in mice with CD-aggravated MASH. Furthermore, inulin supplementation restored the hepatic proteome structure similar to that of non-CD MASH mice, a benefit not observed with oat β-glucan. **Conclusions:** Given our prior findings showing oat β-glucan’s superior ability to enrich gut bacterial species associated with MASH improvement under CD, this study highlights inulin’s unique benefits for colonic architecture and hepatic proteome regulation in CD-aggravated MASH.

## 1. Introduction

Metabolic dysfunction-associated steatohepatitis (MASH), previously referred to as non-alcoholic steatohepatitis, represents the more advanced form of metabolic dysfunction-associated steatotic liver disease (MASLD) [1]. Currently, MASH affects approximately 5% of the global population [2]. Although an anti-MASH medication was approved by the FDA in 2024, reported side effects such as diarrhea and nausea have been reported [3]. Consequently, the exploration and use of naturally derived nutritional supplements with minimal side effects remain a promising avenue for alleviating MASH. Early intervention and personalized treatment for MASLD could be advantageous when they are combined with prebiotic usage [4]. Prebiotics are defined as “a substrate that is selectively utilized by host microorganisms conferring a health benefit” [5]. Prebiotic polysaccharides may prevent and treat MASLD by modulating the gut–liver axis [6]. This axis represents the bidirectional communication between the two organs through the portal vein, systemic circulation, and biliary tract [7]. It has been noted that Western diet consumption disrupts gut barrier integrity, allowing bacteria and endotoxins to translocate from the intestine and trigger liver inflammation [8].

In recent years, conventional prebiotic inulin and novel prebiotic β-glucan have been extensively studied for their potential to ameliorate MASLD. These compounds exhibit multifunctional properties, including the ability to reduce liver injury, oxidative stress, steatosis, inflammation, fibrosis, and gut dysbiosis [9,10]. Inulin is a polysaccharide composed of fructose subunits connected by β-(2,1) glycosidic bonds. It can be found in chicory root, Jerusalem artichoke, and dahlia tubers [11]. Native chicory inulin has been acknowledged by the European Food Safety Authority for maintaining normal defecation [12]. Although β-glucan is a polysaccharide composed of glucose subunits, different chemical structures are found in different sources of β-glucan. Cereal-derived β-glucans possess mixed linkages of β-(1,3) and β-(1,4) glycosidic bonds, whereas yeast and fungal β-glucans feature β-(1,3) and β-(1,6) glycosidic bonds [13]. Approved by the US Food and Drug Administration, oat β-glucan is beneficial for lowering the risk of coronary heart disease [14].

Our previous study has demonstrated that both inulin and oat β-glucan could alleviate circadian-disruption (CD)-aggravated MASH in terms of anti-liver injury, anti-inflammatory, and anti-fibrotic activities [15]. While existing literature demonstrates that these prebiotics can prevent MASH by downregulating pro-inflammatory mediators [9,10], their effects on the colonic architecture and hepatic proteome remain poorly characterized. To address this gap, we aimed to investigate how inulin and oat β-glucan affect colonic architecture and hepatic proteome in CD-aggravated MASH, extending our previous study [15]. Proteomics enables direct insights into the molecular mechanisms underlying disease [16], advancing the way we manage the complicated pathophysiology of MASLD [17]. We hypothesized that mice with CD-aggravated MASH would exhibit worsened colonic architecture and altered hepatic proteome compared to mice with non-CD MASH, while prebiotic supplementation might mitigate these effects. This study brings new insights into how CD and prebiotic supplementation influence the hepatic proteome in MASH, which distinguishes it from earlier reports.

## 2. Materials and Methods

### 2.1. Design of Animal Experiment

An investigation of colonic architecture and hepatic proteome was performed using the distal colon and liver collected from our prior animal experiment [15]. Thirty male C57BL/6J mice (8 to 10 weeks old) were obtained from the Laboratory Animal Services Centre of The Chinese University of Hong Kong (CUHK). The mice were individually housed under controlled conditions (temperature: 21–23 °C; relative humidity: 50–60%; light intensity: 300–400 lx) at the Greenhouse Extension of CUHK. All mice had ad libitum access to water and diet. They were fed either a chow diet (TD.94048, Inotiv, West Lafayette, IN, USA) with water or a MASH-inducing fructose, palmitate, cholesterol, and trans-fat (FPC) diet (TD.160785, Inotiv, West Lafayette, IN, USA) with water containing 42 g/L glucose and fructose (55%/45%, *w*/*w*) [18,19].

Following a week of adaptive feeding using a chow diet, mice were randomly allocated to one of the six groups (*n* = 5/group): non-shifted chow (NSC), non-shifted FPC diet (NSFPC), shifted chow (SC), shifted FPC diet (SFPC), shifted FPC diet with inulin supplementation (SINU), shifted FPC diet with oat β-glucan supplementation (SOBG). Using data from Kanagasabapathy et al., where high-fat diet-fed mice exhibited alanine aminotransferase levels of 48 ± 1.4 mmol/L compared to 45 ± 1.2 mmol/L in normal diet-fed mice (mean difference = 3.0 mmol/L, pooled SD = 1.3 mmol/L, Cohen’s d = 2.31) [20], a power analysis indicated that at least 4 mice per group would be required to achieve statistical significance (*p* < 0.05) with 90% power. The NSC and NSFPC groups lived under a constant light–dark cycle (12 h light/12 h dark) where lights were on from 6 am to 6 pm. The SC, SFPC, SINU, and SOBG groups experienced CD because the light–dark cycle was reversed weekly. The experimental period lasted 26 weeks but prebiotic supplementation (500 mg/kg body weight) was provided via drinking water during the final 10 weeks. The drinking bottles were changed twice a week in a randomized order. Chicory root inulin OraftiHP (purity: ∼100%; average degree of polymerization: ≥23) and oat β-glucan (purity: 80%) were obtained from Beneo (Pemuco, Chile) and Xi’an Sgonek Biological Technology Co. Ltd. (Xi’an, China), respectively.

The body weight and intake of food and fluid were monitored weekly. After 26 weeks, all animals were euthanized by carbon dioxide asphyxiation. Serum, distal colon, and liver samples were harvested and stored at −80 °C for subsequent analysis. All animal experiments were performed in compliance with the guidelines and regulations of the Department of Health and CUHK, with approval from the CUHK Animal Experimentation Ethics Committee (ref no. 21-056-MIS). Data for this animal experiment regarding metabolic phenotypes (body weight, liver weight, liver index, liver enzymes, insulin), liver histopathology, the hepatic expression of circadian clock and inflammatory genes, cecal short-chain fatty acids, and gut microbiota composition and its function could be found in our previous study [15]. No criteria were set to include and exclude animals during the experiment, and data points during the analysis. Additionally, no blinding was performed for group allocation at any experimental stage.

### 2.2. Histological Examination of Colon and Endotoxemia Evaluation

Distal colon tissue was fixed in 10% formalin at 4 °C and subsequently embedded in paraffin. Hematoxylin and eosin (H&E) staining was performed on 5 μm colon sections. Images were captured using a Carl Zeiss PALM Inverted microscope (Oberkochen, Baden-Wurttemberg, Germany). The colonic inflammation infiltration scores were assigned by two gastroenterology experts who assessed the depth of inflammation (0 = no infiltrate; 1 = infiltrate detected at the mucosa; 2 = infiltrate extending to the submucosa; 3 = infiltrate reaching the muscularis propria) [21]. Scoring was based on one image (a defined section displayed at 10× magnification) per mouse. Serum lipopolysaccharide-binding protein (LBP) was used as a measurement for endotoxemia and assessed using a commercial ELISA kit (Abcam, Cambridge, UK). One-way ANOVA followed by Fisher’s LSD test was adopted to identify statistical significance using GraphPad Prism 9 (GraphPad Software, La Jolla, CA, USA). *p*-value below 0.05 was considered statistically significant.

### 2.3. Proteomic Mass Spectrometry Analysis

Liver samples were prepared for proteomic mass spectrometry (MS) analysis using the EasyPep™ Mini MS Sample Prep Kit (Thermo Fisher Scientific, Waltham, MA, USA). MS analysis was performed on the Orbitrap Fusion™ Lumos™ Tribrid™ Mass Spectrometer (Thermo Fisher Scientific, Waltham, MA, USA) coupled with the LC UltiMate 3000 RSLCnano system (Thermo Fisher Scientific, Waltham, MA, USA). Peptides were separated at 50 °C using the C-18 μ-precolumn (300 μm i.d. × 5 mm; Thermo Fisher Scientific, Waltham, MA, USA) and then the Acclaim™ PepMap™ RSLC nanoViper C-18 column (75 μm × 25 cm; Thermo Fisher Scientific, Waltham, MA, USA). Protein separation was carried out at a flow rate of 0.3 μL/min with mobile phase A consisting of 98% H_2_O, 1.9% acetonitrile (ACN), and 0.1% formic acid (FA), and mobile phase B comprising 98% ACN, 1.9% H_2_O, and 0.1% FA. To detect total protein, the following LC gradient was adopted: an initial hold at 100% A lasting 5 min, transitioning from 0% to 6% B in 3 min. The gradient rose to 18% B across 40 min, 30% B during the subsequent 10 min, and 80% B in 2 min. It was maintained at 80% B for 5 min before reverting to 100% A during a 10 min re-equilibration phase.

The data-dependent MS/MS mode was used to configure the Orbitrap, controlled by Xcalibur software (version 4.1; Thermo Fisher Scientific, Waltham, MA, USA). A full-scan spectrum within the 375 to 1500 *m*/*z* range and tandem mass spectra (MS/MS) were acquired. The instrument was calibrated before measurements. Then, it was run in positive mode, with the spray voltage set to 2 kV and the capillary temperature to 300 °C, respectively. The resolution of full scans acquired in the Orbitrap was 60,000 at 400 *m*/*z*. The precursor ion selection was made with an AGC > 4 × 10^5^ and an ion charge > 1. To conduct fragmentation, high-energy collisional dissociation was carried out at the far side of the C-trap (30% normalized collision energy, 1.6 *m*/*z* isolation window, 250 ms maximum injection time, 15,000 Orbitrap resolution).

Data processing and protein identification were performed using the Proteome Discoverer Platform (version 2.4.1; Thermo Fisher Scientific, Waltham, MA, USA) coupled with an in-house SEQUEST server based on these parameters: trypsin as enzyme, 2 maximum missed cleavage, 10 ppm precursor mass tolerance, 0.2 Da fragment mass tolerance, carbamidomethylation of cysteine as static modification, and N-terminal acetylation and oxidation of methionine as dynamic modifications. The raw files were searched against the *Mus musculus* database. Peptides were validated by the Percolator algorithm based on *q*-value < 0.01 and false discovery rate < 0.01. Label-free quantification analysis (*n* = 5/group) was performed for whole-proteome analysis. A principal component analysis (PCA) plot was generated by the Proteome Discoverer Platform (Thermo Fisher Scientific, Waltham, MA, USA) to visualize the distinguishable hepatic proteome structures between groups. Proteins were considered differentially expressed if the abundance ratios showed Benjamini-corrected *p*-values less than 0.05. The total differentially expressed proteins (DEPs) were uploaded to the STRING database (https://string-db.org/; accessed on 5 February 2025) for gene ontology (GO) and Kyoto Encyclopedia of Genes and Genomes (KEGG) pathway enrichment analysis.

## 3. Results and Discussion

### 3.1. Effects of Circadian Disruption and Prebiotic Supplementation on Colon Histopathology and Endotoxemia

**Figure 1:**
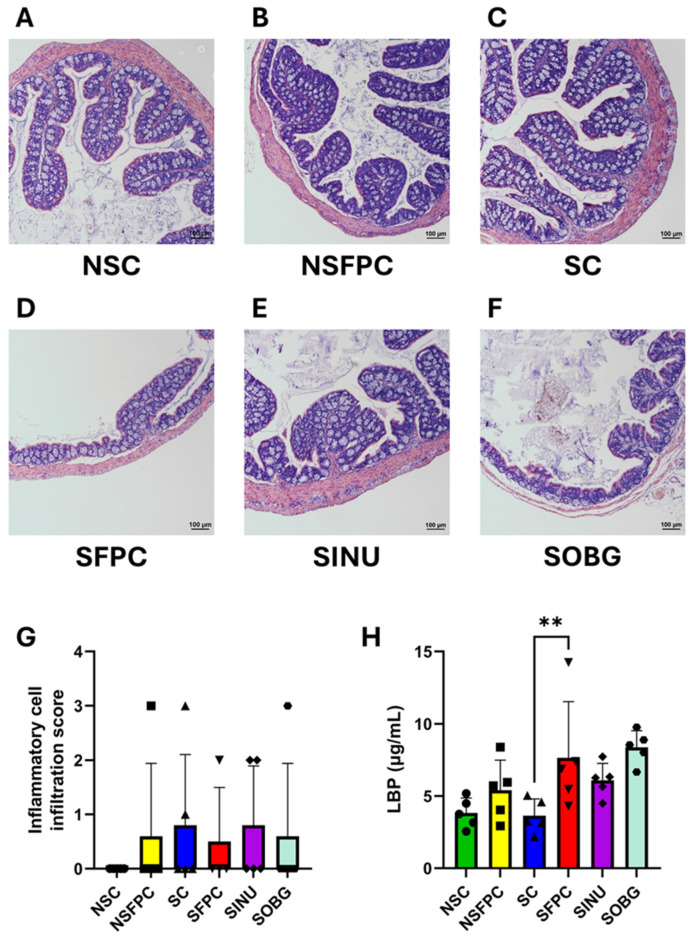
Inulin is more effective than oat β-glucan in improving the colonic architecture. Representative H&E-stained distal colon sections from the (**A**) non-shifted chow (NSC), (**B**) non-shifted FPC diet (NSFPC), (**C**) shifted chow (SC), (**D**) shifted FPC diet (SFPC), (**E**) shifted FPC diet with inulin supplementation (SINU), and (**F**) shifted FPC diet with oat β-glucan supplementation (SOBG) groups. Scale bar: 100 μm. (**G**) Inflammatory cell infiltration score. *n* = 4–5 per group. (**H**) Serum lipopolysaccharide-binding protein (LBP) levels. *n* = 5 per group. Data are shown as mean ± SD. **: *p* < 0.01.

Normal colon architecture with well-organized crypts was shown in the NSC, NSFPC, and SC groups (Figure 1A–C). However, CD worsened the colonic architecture in MASH mice, as shown by severe crypt loss and flattening in the SFPC group (Figure 1D). Greater restoration of colonic structure was demonstrated by the SINU group compared to the SOBG group, as evidenced by better crypt organization (Figure 1E,F). A comparable degree of colonic inflammation was found in all groups except the NSC group (Figure 1G). The levels of serum LBP were significantly increased in the SFPC group compared to the SC group (Figure 1H). However, colonic inflammation and endotoxemia were not alleviated after 10 weeks of prebiotic supplementation. Previously, it was also demonstrated that serum lipopolysaccharide (LPS) levels were not significantly altered after treating the MASLD mice with Jerusalem artichoke inulin for eight weeks, despite an improvement in MASLD indicators being seen [22]. Since decreased levels of endotoxemia (represented by circulatory LPS) resulting from inulin supplementation were only observed in MASLD prevention studies [10], the timing of prebiotic supplementation to manage MASLD is critical. Therefore, it is suggested that early prebiotic supplementation would be more significant and useful to prevent MASH-associated endotoxemia.

Our H&E staining results demonstrate that CD deteriorates colonic architecture in MASH mice. This aligns with previous studies showing that constant light exposure (a CD model) significantly reduced the expression of key gut barrier markers occludin and zonula occludens-1 in high-fat diet-fed MASH mice based on immunohistochemical analysis, as well as increased levels of serum LPS and liver LBP mRNA [23]. The beneficial effect of improving colonic architecture has not been revealed in the prior prevention or treatment studies of β-glucan and treatment studies of inulin in the field of MASLD [9,10]. More importantly, our findings shed light on the potential mechanism underlying the anti-MASH effects of these prebiotics, particularly under CD conditions [15].

### 3.2. Effects of Circadian Disruption and Prebiotic Supplementation on Hepatic Proteome Structure

**Figure 2:**
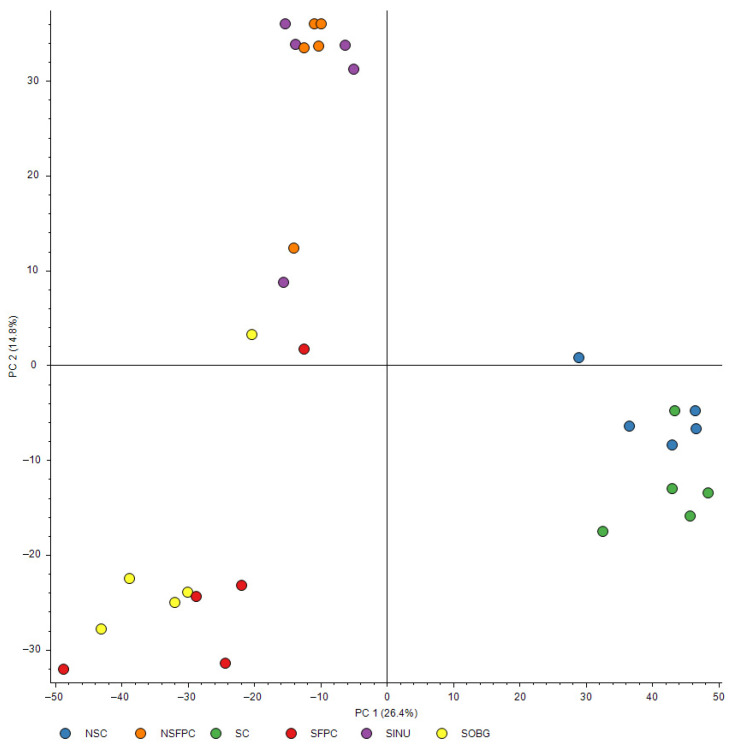
PCA of hepatic proteome structure. Different colors of dots represent different groups of mice. Blue: non-shifted chow (NSC); orange: non-shifted FPC diet (NSFPC); green: shifted chow (SC); red: shifted FPC diet (SFPC); purple: shifted FPC diet with inulin supplementation (SINU); yellow: shifted FPC diet with oat β-glucan supplementation (SOBG).

To the best of our knowledge, using proteomic profiling is a novel approach to study the effects of CD and prebiotic supplementation on MASH. Three distinct clusters were observed in the PCA of the proteome structure (Figure 2). The NSC and SC groups clustered closely together, indicating that CD minimally affects the hepatic proteome structure in mice fed a chow diet. In contrast, the NSFPC and SFPC groups formed separate clusters, highlighting the significant impact of CD on the hepatic proteome in MASH. No significant difference in hepatic proteome structure was found between the SFPC and SOBG groups as they clustered together. Interestingly, the SINU group clustered with the NSFPC group rather than the SOBG group.

These findings suggest that inulin has a stronger modulatory effect than oat β-glucan in shifting the hepatic proteome structure of CD-aggravated MASH mice toward a pattern resembling that of the non-CD MASH. This aligns with our previous findings showing inulin’s superior efficacy in restoring the expression of key hepatic circadian clock genes (*Clock* and *Bmal1*) toward levels observed in the SC group [15]. Although both prebiotics exhibited similar anti-MASH activity as shown previously [15], the enhanced ability of inulin to restore circadian clock gene expression may underlie its greater impact on proteome restoration. This suggests that inulin may play a more prominent role in mitigating CD-induced hepatic proteome disturbances. This study is the first to provide an overview of the effects of CD and prebiotic supplementation on the hepatic proteome structure, offering valuable insights into potential dietary strategies for combating MASH-related hepatic proteome disturbances. Since prebiotic supplementation was not conducted in non-CD MASH and normal chow groups in this study, whether the beneficial effects of inulin are specific to CD and generalizable remains to be determined.

### 3.3. Comparative Analysis of Hepatic Proteome

The number of total DEPs found in each group comparison is shown in Table 1. Complete lists of total DEPs for all comparisons are provided in Supplementary Tables S1–S6. The top 20 most significantly enriched GO terms for biological process, molecular function, and cellular component are presented in Supplementary Figures S1–S3.

**Figure 3:**
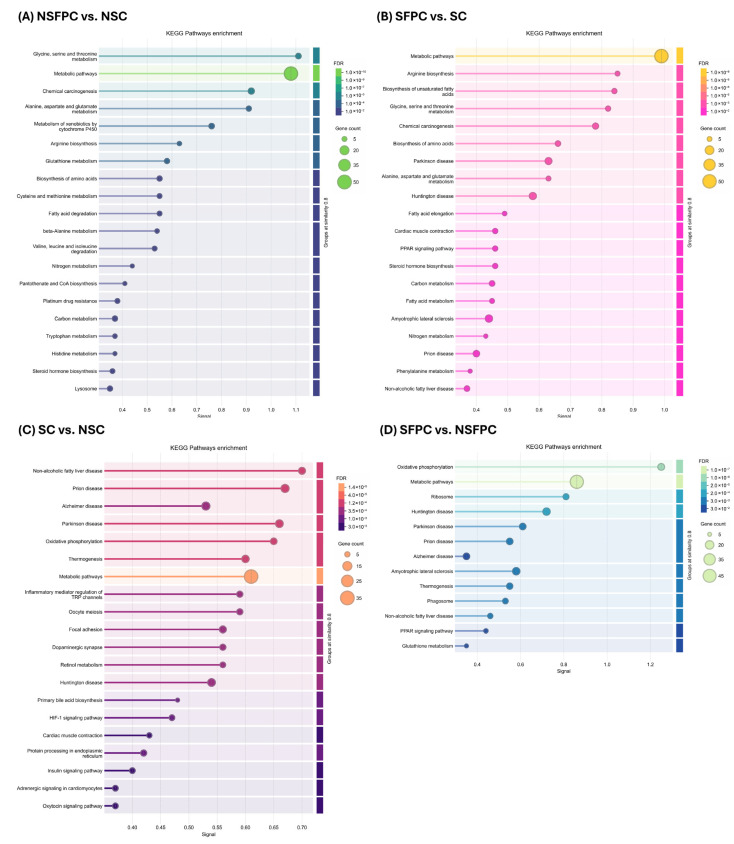

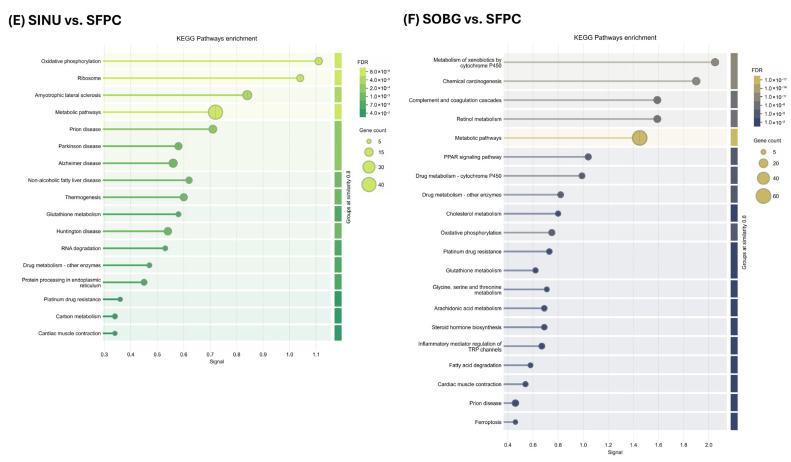
KEGG pathways enrichment analysis of total differentially expressed proteins in different group comparisons. (**A**) NSFPC vs. NSC. (**B**) SFPC vs. SC. (**C**) SC vs. NSC. (**D**) SFPC vs. NSFPC. (**E**) SINU vs. SFPC. (**F**) SOBG vs. SFPC. FDR: false discovery rate; NSC: non-shifted chow; NSFPC: non-shifted FPC diet; SC: shifted chow; SFPC: shifted FPC diet; SINU: shifted FPC diet with inulin supplementation; SOBG: shifted FPC diet with oat β-glucan supplementation.

KEGG pathway enrichment analysis revealed that nine of the top 20 pathways were commonly affected in the NSFPC and SFPC groups compared to their respective controls (NSC and SC). These pathways included (1) glycine, serine, and threonine metabolism; (2) metabolic pathways; (3) chemical carcinogenesis, (4) alanine, aspartate, and glutamate metabolism; (5) arginine biosynthesis; (6) biosynthesis of amino acids; (7) nitrogen metabolism; (8) carbon metabolism; and (9) steroid hormone biosynthesis (Figure 3A,B). A previous transcriptomic study also reported that high-fat diet-induced MASLD significantly altered pathways related to alanine, aspartate, and glutamate metabolism; glycine, serine, and threonine metabolism; chemical carcinogenesis; carbon metabolism; and steroid hormone biosynthesis in rat liver [24]. Additionally, the non-alcoholic fatty liver disease pathway was significantly affected in the SFPC group compared to the SC group (Figure 3B).

Furthermore, CD commonly altered eight pathways regardless of the type of diet consumed, including (1) non-alcoholic fatty liver disease; (2) prion disease; (3) Alzheimer’s disease; (4) Parkinson’s disease; (5) oxidative phosphorylation; (6) thermogenesis; (7) metabolic pathways; and (8) Huntington disease (Figure 3C,D). A previous study on hypothalamic differentially expressed genes in mice with circadian disturbances also identified oxidative phosphorylation, Alzheimer’s disease, and prion disease as significantly affected pathways [25]. Since oxidative phosphorylation and thermogenesis are known to be regulated by the circadian clock [26,27], our findings suggest that CD may have undesirable effects on these pathways.

Interestingly, the same eight pathways were influenced by inulin supplementation (Figure 3E), potentially driving the SINU group toward a hepatic proteomic profile similar to the NSFPC group (Figure 2). Additionally, pathways related to prion disease, PPAR signaling pathway, oxidative phosphorylation, and glutathione metabolism, which were significantly affected in the SFPC group compared to the NSFPC group (Figure 3D), were regulated by oat β-glucan supplementation (Figure 3F). Moreover, oxidative phosphorylation, amyotrophic lateral sclerosis, metabolic pathways, prion disease, glutathione metabolism, drug metabolism—other enzymes, platinum drug resistance, and cardiac muscle contraction were the commonly affected pathways in the SINU and SOBG groups when compared to the SFPC group (Figure 3E,F). Oat β-glucan is well-known for its cholesterol-lowering effects [28], and the alteration of cholesterol metabolism in the SOBG group was also confirmed in this study (Figure 3F).

Despite the limited literature on the effects of prebiotics inulin and oat β-glucan on hepatic proteome in the context of CD and MASH, our findings demonstrated the potential of these prebiotics in alleviating the CD- and MASH diet-induced hepatic proteome disturbances. To gain an overview of the significantly enriched KEGG pathways identified through comparative analysis, Table 2 displays pathways unique to individual group comparisons, and Table 3 shows those shared across multiple group comparisons.

## 4. Conclusions

This study provides novel evidence of how prebiotics inulin and oat β-glucan differentially affect colonic architecture and hepatic proteome in CD-aggravated MASH. Our findings confirm that CD worsens colonic architecture and alters the hepatic proteome in MASH. Additionally, we demonstrate that inulin might be more effective than oat β-glucan in improving colonic architecture and restoring the hepatic proteome toward a non-CD MASH state. However, the distinct ability of oat β-glucan to enrich gut microbial species associated with MASH improvement, which was not observed with inulin, underscores its complementary role [15]. Therefore, further investigation of the combined supplementation of both prebiotics would be useful to understand whether comprehensive benefits for MASH recovery could be achieved.

Some limitations should be noted, including small sample size, the exclusive use of male mice, the absence of gut permeability assays, and reliance on archived tissues from our previous study [15]. In future studies, exploring the effects of prebiotic supplementation in female mice would be beneficial to enhance the study’s generalizability. To directly evaluate the impact of prebiotics on gut barrier function, gut permeability assays (e.g., FITC-dextran) could be included. Our study identifies distinct prebiotic-specific effects essential for accelerating the development of precision nutrition strategies to manage MASLD. The application of proteomic profiling enables mechanistically informed prebiotic supplementation. Our findings could guide the design of tailored prebiotic and synbiotic formulations for MASLD management, particularly in populations exposed to CD. Additionally, this study highlights the importance of prebiotic supplementation as a dietary practice to ameliorate CD-aggravated MASH.

## Figures and Tables

**Table 1:** The number of total differentially expressed proteins (DEPs) identified in various group comparisons.

Group Comparison	No. of Total DEPs
NSFPC vs. NSC	213
SFPC vs. SC	242
SC vs. NSC	191
SFPC vs. NSFPC	246
SINU vs. SFPC	226
SOBG vs. SFPC	230

**Table 2:** KEGG pathways uniquely enriched in each group comparison.

NSFPC vs. NSC	SFPC vs. SC	SC vs. NSC	SFPC vs. NSFPC	SINU vs. SFPC	SOBG vs. SFPC
Cysteine and methionine metabolism	Biosynthesis of unsaturated fatty acids	Adrenergic signaling in cardiomyocytes	Phagosome	RNA degradation	Arachidonic acid metabolism
Histidine metabolism	Fatty acid elongation	Dopaminergic synapse	/	/	Cholesterol metabolism
Lysosome	Fatty acid metabolism	Focal adhesion	/	/	Complement and coagulation cascades
Pantothenate and CoA biosynthesis	Phenylalanine metabolism	HIF-1 signaling pathway	/	/	Drug metabolism—cytochrome P450
Tryptophan metabolism	/	Insulin signaling pathway	/	/	Ferroptosis
Valine, leucine, and isoleucine degradation	/	Oocyte meiosis	/	/	/
beta-Alanine metabolism	/	Oxytocin signaling pathway	/	/	/
/	/	Primary bile acid biosynthesis	/	/	/

**Table 3:** KEGG pathways shared across multiple group comparisons.

NSFPC vs. NSC	SFPC vs. SC	SC vs. NSC	SFPC vs. NSFPC	SINU vs. SFPC	SOBG vs. SFPC
/	/	/	/	Drug metabolism—other enzymes	Drug metabolism—other enzymes
/	/	/	Ribosome	Ribosome	/
/	/	Alzheimer disease	Alzheimer disease	Alzheimer disease	/
/	/	Inflammatory mediator regulation of TRP channels	/	/	Inflammatory mediator regulation of TRP channels
/	/	Oxidative phosphorylation	Oxidative phosphorylation	Oxidative phosphorylation	Oxidative phosphorylation
/	/	Protein processing in endoplasmic reticulum	/	Protein processing in endoplasmic reticulum	/
/	/	Retinol metabolism	/	/	Retinol metabolism
/	/	Thermogenesis	Thermogenesis	Thermogenesis	/
/	Amyotrophic lateral sclerosis	/	Amyotrophic lateral sclerosis	Amyotrophic lateral sclerosis	/
/	Cardiac muscle contraction	Cardiac muscle contraction	/	Cardiac muscle contraction	Cardiac muscle contraction
/	Huntington disease	Huntington disease	Huntington disease	Huntington disease	/
/	Non-alcoholic fatty liver disease	Non-alcoholic fatty liver disease	Non-alcoholic fatty liver disease	Non-alcoholic fatty liver disease	/
/	PPAR signaling pathway	/	PPAR signaling pathway	/	PPAR signaling pathway
/	Parkinson disease	Parkinson disease	Parkinson disease	Parkinson disease	/
/	Prion disease	Prion disease	Prion disease	Prion disease	Prion disease
Alanine, aspartate, and glutamate metabolism	Alanine, aspartate, and glutamate metabolism	/	/	/	/
Arginine biosynthesis	Arginine biosynthesis	/	/	/	/
Biosynthesis of amino acids	Biosynthesis of amino acids	/	/	/	/
Carbon metabolism	Carbon metabolism	/	/	Carbon metabolism	/
Chemical carcinogenesis	Chemical carcinogenesis	/	/	/	Chemical carcinogenesis
Fatty acid degradation	/	/	/	/	Fatty acid degradation
Glutathione metabolism	/	/	Glutathione metabolism	Glutathione metabolism	Glutathione metabolism
Glycine, serine, and threonine metabolism	Glycine, serine, and threonine metabolism	/	/	/	Glycine, serine, and threonine metabolism
Metabolic pathways	Metabolic pathways	Metabolic pathways	Metabolic pathways	Metabolic pathways	Metabolic pathways
Metabolism of xenobiotics by cytochrome P450	/	/	/	/	Metabolism of xenobiotics by cytochrome P450
Nitrogen metabolism	Nitrogen metabolism	/	/	/	/
Platinum drug resistance	/	/	/	Platinum drug resistance	Platinum drug resistance
Steroid hormone biosynthesis	Steroid hormone biosynthesis	/	/	/	Steroid hormone biosynthesis

## Data Availability

The original contributions presented in this study are included in the article/Supplementary Material. Further inquiries can be directed to the corresponding authors.

## Supplementary Materials

The following supporting information can be downloaded at: https://www.mdpi.com/article/10.3390/nu17132245/s1, Figure S1: Biological process (gene ontology) enrichment analysis of total differentially expressed proteins in different group comparisons. Figure S2: Molecular function (gene ontology) enrichment analysis of total differentially expressed proteins in different group comparisons. Figure S3: Cellular component (gene ontology) enrichment analysis of total differentially expressed proteins in different group comparisons; Table S1: List of total differentially expressed proteins (NSFPC vs. NSC); Table S2: List of total differentially expressed proteins (SFPC vs. SC); Table S3: List of total differentially expressed proteins (SC vs. NSC); Table S4: List of total differentially expressed proteins (SFPC vs. NSFPC); Table S5: List of total differentially expressed proteins (SINU vs. SFPC); Table S6: List of total differentially expressed proteins (SOBG vs. SFPC).

## Abbreviations

The following abbreviations are used in this manuscript:

CD	Circadian disruption
DEPs	Differentially expressed proteins
FPC	Fructose, palmitate, cholesterol, and trans-fat
MASH	Metabolic dysfunction-associated steatohepatitis
MASLD	Metabolic dysfunction-associated steatotic liver disease
NSC	Non-shifted chow
NSFPC	Non-shifted FPC diet
PCA	Principal component analysis
SC	Shifted chow
SFPC	Shifted FPC diet
SINU	Shifted FPC diet with inulin supplementation
SOBG	Shifted FPC diet with oat β-glucan supplementation

## References

[1] Do A., Zahrawi F., Mehal W.Z. (2025). Therapeutic landscape of metabolic dysfunction-associated steatohepatitis (MASH). Nat. Rev. Drug Discov..

[2] Miao L., Targher G., Byrne C.D., Cao Y.Y., Zheng M.H. (2024). Current status and future trends of the global burden of MASLD. Trends Endocrinol. Metab..

[3] FDA FDA Approves First Treatment for Patients with Liver Scarring Due to Fatty Liver Disease. https://www.fda.gov/news-events/press-announcements/fda-approves-first-treatment-patients-liver-scarring-due-fatty-liver-disease.

[4] Wang S., Zhang R., Guo P., Yang H., Liu Y., Zhu H. (2025). Association of prebiotic/probiotic intake with MASLD: Evidence from NHANES and randomized controlled trials in the context of prediction, prevention, and a personalized medicine framework. EPMA J..

[5] Gibson G.R., Hutkins R., Sanders M.E., Prescott S.L., Reimer R.A., Salminen S.J., Scott K., Stanton C., Swanson K.S., Cani P.D. (2017). Expert consensus document: The International Scientific Association for Probiotics and Prebiotics (ISAPP) consensus statement on the definition and scope of prebiotics. Nat. Rev. Gastroenterol. Hepatol..

[6] Guo Q., Li Y., Dai X., Wang B., Zhang J., Cao H. (2023). Polysaccharides: The potential prebiotics for metabolic associated fatty liver disease (MAFLD). Nutrients.

[7] Han H., Jiang Y., Wang M., Melaku M., Liu L., Zhao Y., Everaert N., Yi B., Zhang H. (2023). Intestinal dysbiosis in nonalcoholic fatty liver disease (NAFLD): Focusing on the gut-liver axis. Crit. Rev. Food Sci. Nutr..

[8] Bauer K.C., Littlejohn P.T., Ayala V., Creus-Cuadros A., Finlay B.B. (2022). Nonalcoholic fatty liver disease and the gut-liver axis: Exploring an undernutrition perspective. Gastroenterology.

[9] Kei N., Wong V.W.S., Lauw S., You L., Cheung P.C.K. (2023). Utilization of food-derived β-glucans to prevent and treat non-alcoholic fatty liver disease (NAFLD). Foods.

[10] Kei N., Lauw S., Wong V.W.S., Cheung P.C.K. (2024). A mini-review on prebiotic inulin to prevent and treat non-alcoholic fatty liver disease. Food Biosci..

[11] Karimi I., Ghowsi M., Mohammed L.J., Haidari Z., Nazari K., Schioth H.B. (2025). Inulin as a biopolymer; Chemical structure, anticancer effects, nutraceutical potential and industrial applications: A comprehensive review. Polymers.

[12] EFSA Panel on Dietetic Products, Nutrition and Allergies (NDA) (2015). Scientific opinion on the substantiation of a health claim related to “native chicory inulin” and maintenance of normal defecation by increasing stool frequency pursuant to Article 13.5 of Regulation (EC) No 1924/2006. EFSA J..

[13] Singla A., Gupta O.P., Sagwal V., Kumar A., Patwa N., Mohan N., Ankush, Kumar D., Vir O., Singh J. (2024). Beta-glucan as a soluble dietary fiber source: Origins, biosynthesis, extraction, purification, structural characteristics, bioavailability, biofunctional attributes, industrial utilization, and global trade. Nutrients.

[14] Mathews R., Kamil A., Chu Y. (2020). Global review of heart health claims for oat beta-glucan products. Nutr. Rev..

[15] Kei N., Cheung K.K., Ma K.L., Yau T.K., Lauw S., Wong V.W.S., You L., Cheung P.C.K. (2024). Effects of oat β-glucan and inulin on alleviation of nonalcoholic steatohepatitis aggravated by circadian disruption in C57BL/6J mice. J. Agric. Food Chem..

[16] Pirola C.J., Fernandez Gianotti T., Sookoian S. (2025). The proteomics of MASLD progression: Insights from functional analysis to drive the development of new therapeutic solutions. Aliment. Pharmacol. Ther..

[17] Hernandez R., Garcia-Rodriguez N.S., Arriaga M.A., Perez R., Bala A.A., Leandro A.C., Diego V.P., Almeida M., Parsons J.G., Manusov E.G. (2025). The hepatocellular model of fatty liver disease: From current imaging diagnostics to innovative proteomics technologies. Front. Med..

[18] Zhu C., Kim K., Wang X., Bartolome A., Salomao M., Dongiovanni P., Meroni M., Graham M.J., Yates K.P., Diehl A.M. (2018). Hepatocyte Notch activation induces liver fibrosis in nonalcoholic steatohepatitis. Sci. Transl. Med..

[19] Wang X., Zheng Z., Caviglia J.M., Corey K.E., Herfel T.M., Cai B., Masia R., Chung R.T., Lefkowitch J.H., Schwabe R.F. (2016). Hepatocyte TAZ/WWTR1 promotes inflammation and fibrosis in nonalcoholic steatohepatitis. Cell Metab..

[20] Kanagasabapathy G., Malek S.N., Mahmood A.A., Chua K.H., Vikineswary S., Kuppusamy U.R. (2013). Beta-glucan-rich extract from *Pleurotus sajor-caju* (Fr.) Singer prevents obesity and oxidative stress in C57BL/6J mice fed on a high-fat diet. Evid.-Based Complement. Alternat. Med..

[21] Wang S., Kang X., Alenius H., Wong S.H., Karisola P., El-Nezami H. (2022). Oral exposure to Ag or TiO_2_ nanoparticles perturbed gut transcriptome and microbiota in a mouse model of ulcerative colitis. Food Chem. Toxicol..

[22] Li J., Jia S., Yuan C., Yu B., Zhang Z., Zhao M., Liu P., Li X., Cui B. (2022). Jerusalem artichoke inulin supplementation ameliorates hepatic lipid metabolism in type 2 diabetes mellitus mice by modulating the gut microbiota and fecal metabolome. Food Funct..

[23] Wei L., Yue F., Xing L., Wu S., Shi Y., Li J., Xiang X., Lam S.M., Shui G., Russell R. (2020). Constant light exposure alters gut microbiota and promotes the progression of steatohepatitis in high fat diet rats. Front. Microbiol..

[24] Yang W., He Y., Liu S., Gan L., Zhang Z., Wang J., Liang J., Dong Y., Wang Q., Hou Z. (2016). Integrative transcriptomic analysis of NAFLD animal model reveals dysregulated genes and pathways in metabolism. Gene.

[25] Zhang Y., Cheng L., Liu Y., Zhang R., Wu Z., Cheng K., Zhang X. (2022). Omics analyses of intestinal microbiota and hypothalamus clock genes in circadian disturbance model mice fed with green tea polyphenols. J. Agric. Food Chem..

[26] Kim J., Sun W. (2024). Circadian coordination: Understanding interplay between circadian clock and mitochondria. Anim. Cells Syst..

[27] Peng X., Chen Y. (2023). The emerging role of circadian rhythms in the development and function of thermogenic fat. Front. Endocrinol..

[28] Joyce S.A., Kamil A., Fleige L., Gahan C.G.M. (2019). The cholesterol-lowering effect of oats and oat beta glucan: Modes of action and potential role of bile acids and the microbiome. Front. Nutr..

