# Supplementary material for: Effects of Prebiotics Inulin and Oat β-Glucan on Colonic Architecture and Hepatic Proteome in Mice with Circadian-Disruption-Aggravated Metabolic Dysfunction-Associated Steatohepatitis
Source: Nutrients. 2025 Jul 5;17(13):2245. doi: 10.3390/nu17132245 (PMC12251635; doi:10.3390/nu17132245)
Supplement: Supplementary file 1 [file nutrients-17-02245-s001.zip › nutrients-3677005-supplementary.pdf]

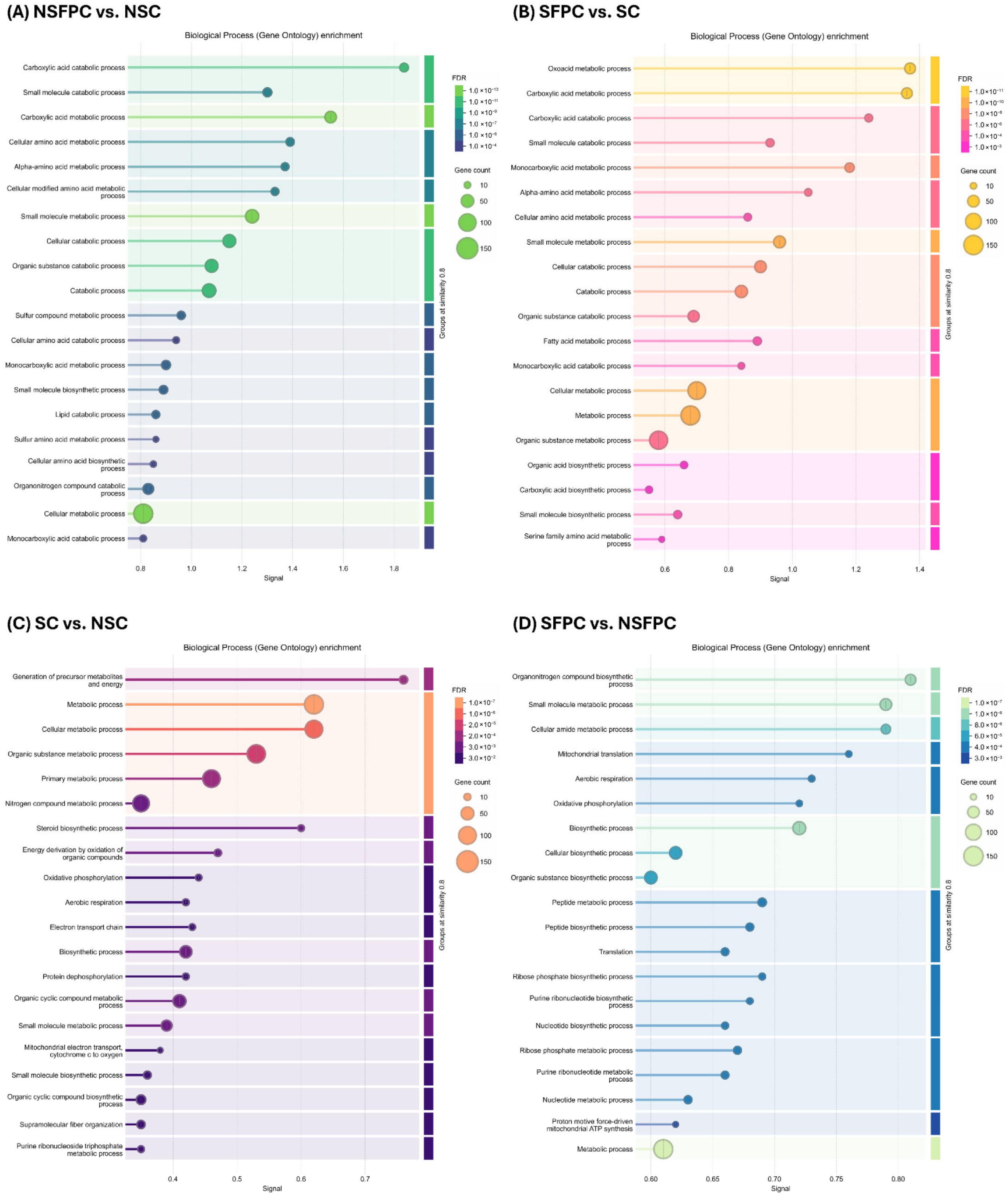

(A) NSFPC vs. NSC
Biological Process (Gene Ontology) enrichment
Carboxylic acid catabolic process
Small molecule catabolic process
Carboxylic acid metabolic process
Cellular amino acid metabolic process
Alpha-amino acid metabolic process
Cellular modified amino acid metabolic process
Small molecule metabolic process
Cellular catabolic process
Organic substance catabolic process
Catabolic process
Sulfur compound metabolic process
Cellular amino acid catabolic process
Monocarboxylic acid metabolic process
Small molecule biosynthetic process
Lipid catabolic process
Sulfur amino acid metabolic process
Cellular amino acid biosynthetic process
Organonitrogen compound catabolic process
Cellular metabolic process
Monocarboxylic acid catabolic process
0.8
1.0
1.2
1.4
1.6
1.8
Signal
Groups at similarity 0.8
FDR
Gene count
10
50
100
150
(B) SFPC vs. SC
Biological Process (Gene Ontology) enrichment
Oxoacid metabolic process
Carboxylic acid metabolic process
Carboxylic acid catabolic process
Small molecule catabolic process
Monocarboxylic acid metabolic process
Alpha-amino acid metabolic process
Cellular amino acid metabolic process
Small molecule metabolic process
Cellular catabolic process
Catabolic process
Organic substance catabolic process
Fatty acid metabolic process
Monocarboxylic acid catabolic process
Cellular metabolic process
Metabolic process
Organic substance metabolic process
Organic acid biosynthetic process
Carboxylic acid biosynthetic process
Small molecule biosynthetic process
Serine family amino acid metabolic process
0.6
0.8
1.0
1.2
1.4
Signal
Groups at similarity 0.8
FDR
Gene count
10
50
100
150
(C) SC vs. NSC
Biological Process (Gene Ontology) enrichment
Generation of precursor metabolites and energy
Metabolic process
Cellular metabolic process
Organic substance metabolic process
Primary metabolic process
Nitrogen compound metabolic process
Steroid biosynthetic process
Energy derivation by oxidation of organic compounds
Oxidative phosphorylation
Aerobic respiration
Electron transport chain
Biosynthetic process
Protein dephosphorylation
Organic cyclic compound metabolic process
Small molecule metabolic process
Mitochondrial electron transport, cytochrome c to oxygen
Small molecule biosynthetic process
Organic cyclic compound biosynthetic process
Supramolecular fiber organization
Purine ribonucleoside triphosphate metabolic process
0.4
0.5
0.6
0.7
Signal
Groups at similarity 0.8
FDR
Gene count
10
50
100
150
(D) SFPC vs. NSFPC
Biological Process (Gene Ontology) enrichment
Organonitrogen compound biosynthetic process
Small molecule metabolic process
Cellular amide metabolic process
Mitochondrial translation
Aerobic respiration
Oxidative phosphorylation
Biosynthetic process
Cellular biosynthetic process
Organic substance biosynthetic process
Peptide metabolic process
Peptide biosynthetic process
Translation
Ribose phosphate biosynthetic process
Purine ribonucleotide biosynthetic process
Nucleotide biosynthetic process
Ribose phosphate metabolic process
Purine ribonucleotide metabolic process
Nucleotide metabolic process
Proton motive force-driven mitochondrial ATP synthesis
Metabolic process
0.60
0.65
0.70
0.75
0.80
Signal
Groups at similarity 0.8
FDR
Gene count
10
50
100
150

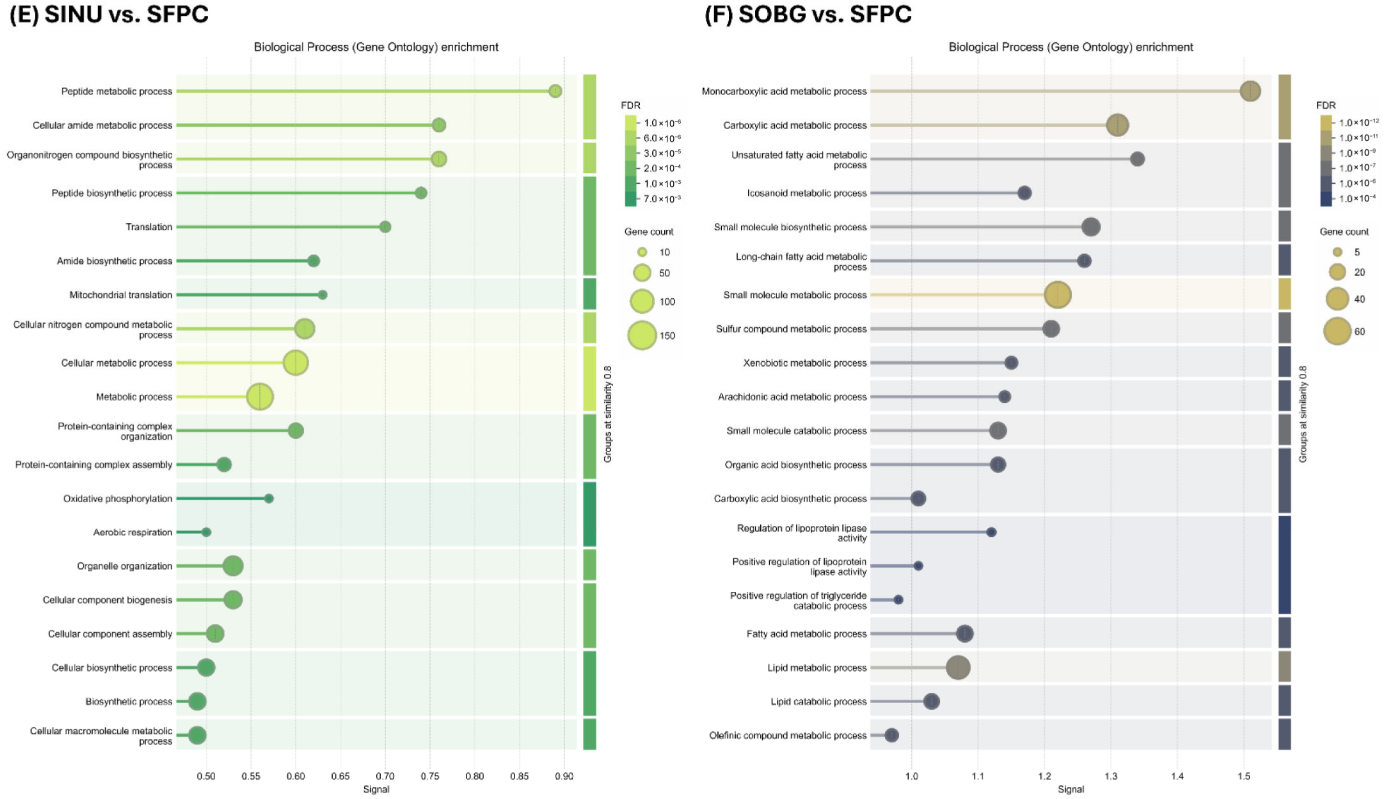

Figure S1. Biological process (gene ontology) enrichment analysis of total differentially expressed proteins in different group comparisons. (A) NSFPC vs. NSC. (B) SFPC vs. SC. (C) SC vs. NSC. (D) SFPC vs. NSFPC. (E) SINU vs. SFPC. (F) SOBG vs. SFPC. FDR: false discovery rate; NSC: non-shifted chow; NSFPC: non-shifted FPC diet; SC: shifted chow; SFPC: shifted FPC diet; SINU: shifted FPC diet with inulin supplementation; SOBG: shifted FPC diet with oat β-glucan supplementation.

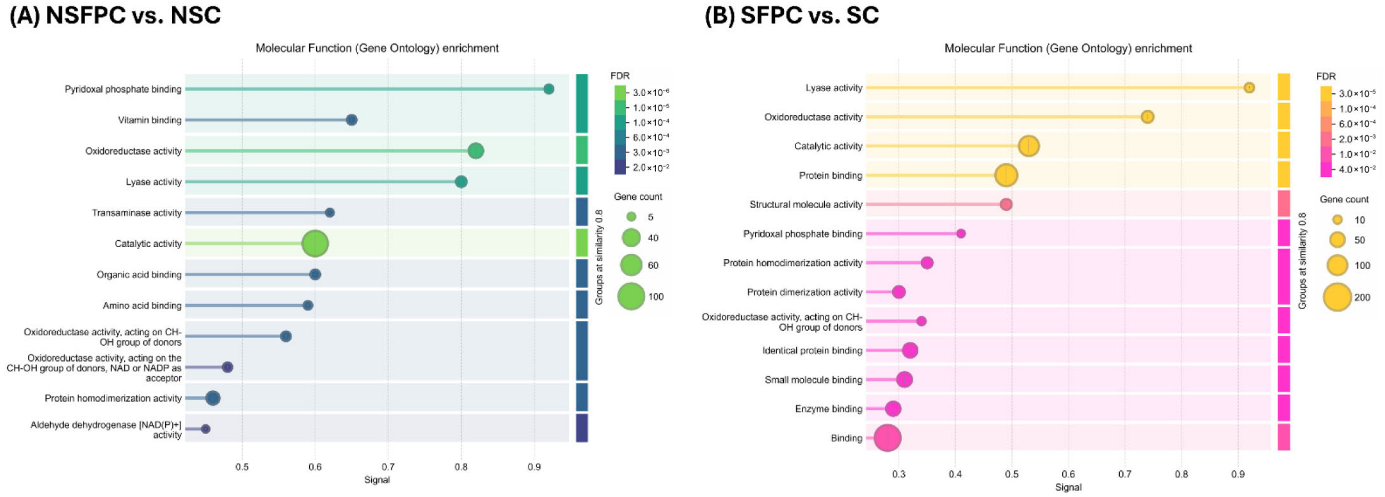

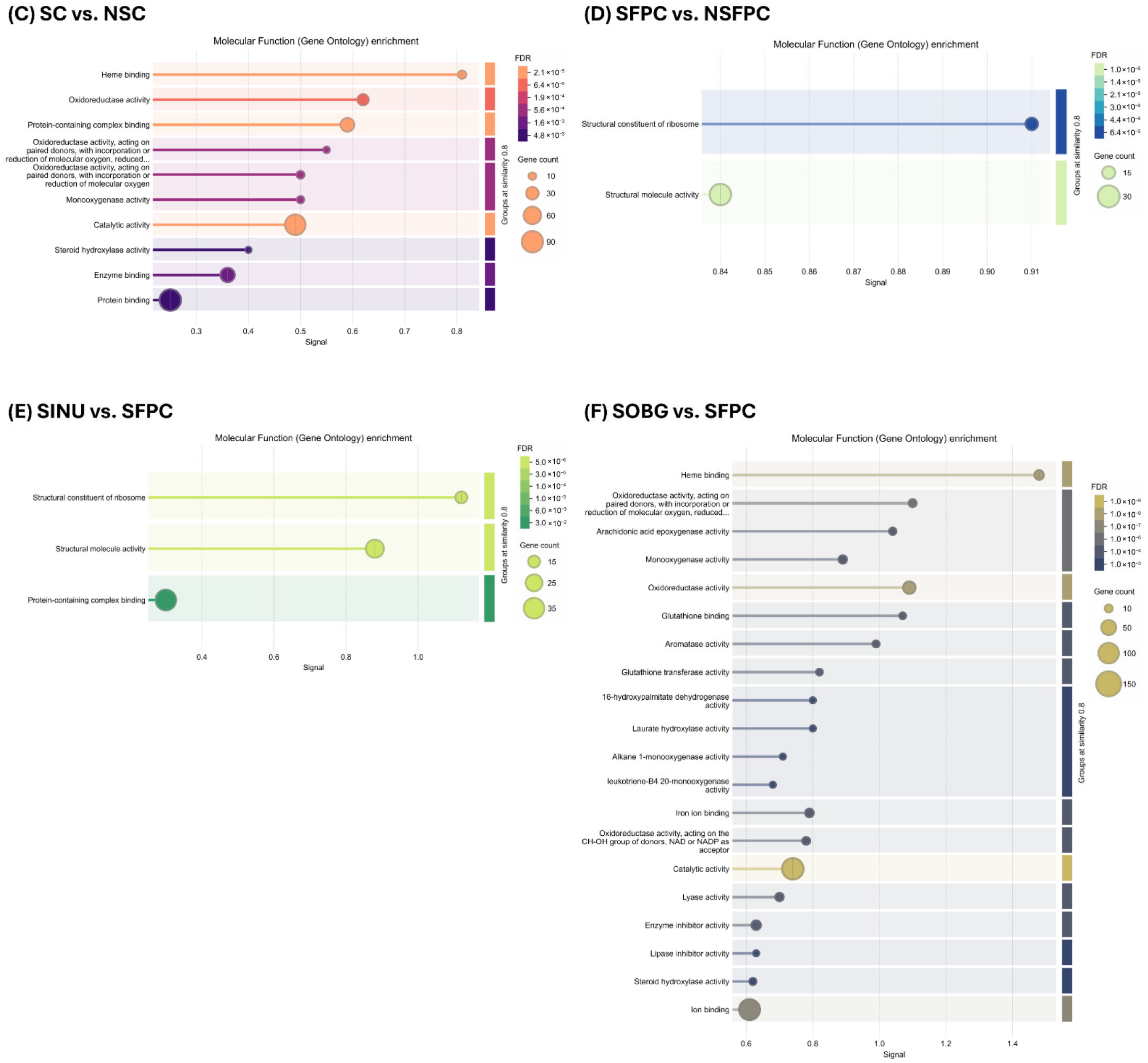

Figure S2. Molecular function (gene ontology) enrichment analysis of total differentially expressed proteins in different group comparisons. (A) NSFPC vs. NSC. (B) SFPC vs. SC. (C) SC vs. NSC. (D) SFPC vs. NSFPC. (E) SINU vs. SFPC. (F) SOBG vs. SFPC. FDR: false discovery rate; NSC: non-shifted chow; NSFPC: non-shifted FPC diet; SC: shifted chow; SFPC: shifted FPC diet; SINU: shifted FPC diet with inulin supplementation; SOBG: shifted FPC diet with oat β-glucan supplementation.

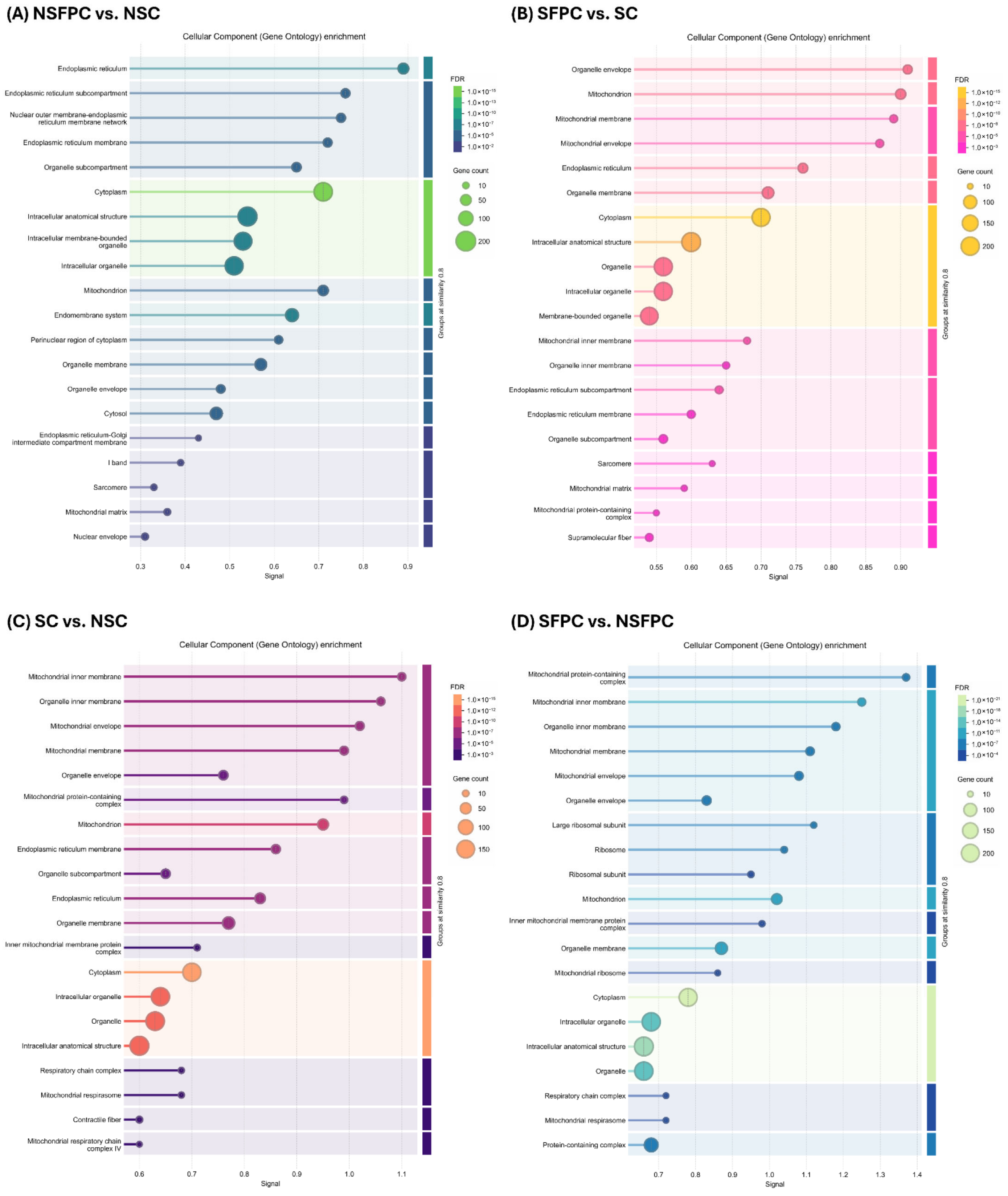
(A) NSFPC vs. NSC
Cellular Component (Gene Ontology) enrichment
Endoplasmic reticulum
Endoplasmic reticulum subcompartment
Nuclear outer membrane-endoplasmic reticulum membrane network
Endoplasmic reticulum membrane
Organelle subcompartment
Cytoplasm
Intracellular anatomical structure
Intracellular membrane-bounded organelle
Intracellular organelle
Mitochondrion
Endomembrane system
Perinuclear region of cytoplasm
Organelle membrane
Organelle envelope
Cytosol
Endoplasmic reticulum-Golgi intermediate compartment membrane
I band
Sarcomere
Mitochondrial matrix
Nuclear envelope
Signal
Groups at similarity 0.8
FDR
Gene count
(B) SFPC vs. SC
Cellular Component (Gene Ontology) enrichment
Organelle envelope
Mitochondrion
Mitochondrial membrane
Mitochondrial envelope
Endoplasmic reticulum
Organelle membrane
Cytoplasm
Intracellular anatomical structure
Organelle
Intracellular organelle
Membrane-bounded organelle
Mitochondrial inner membrane
Organelle inner membrane
Endoplasmic reticulum subcompartment
Endoplasmic reticulum membrane
Organelle subcompartment
Sarcomere
Mitochondrial matrix
Mitochondrial protein-containing complex
Supramolecular fiber
Signal
Groups at similarity 0.8
FDR
Gene count
(C) SC vs. NSC
Cellular Component (Gene Ontology) enrichment
Mitochondrial inner membrane
Organelle inner membrane
Mitochondrial envelope
Mitochondrial membrane
Organelle envelope
Mitochondrial protein-containing complex
Mitochondrion
Endoplasmic reticulum membrane
Organelle subcompartment
Endoplasmic reticulum
Organelle membrane
Inner mitochondrial membrane protein complex
Cytoplasm
Intracellular organelle
Organelle
Intracellular anatomical structure
Respiratory chain complex
Mitochondrial respirasome
Contractile fiber
Mitochondrial respiratory chain complex IV
Signal
Groups at similarity 0.8
FDR
Gene count
(D) SFPC vs. NSFPC
Cellular Component (Gene Ontology) enrichment
Mitochondrial protein-containing complex
Mitochondrial inner membrane
Organelle inner membrane
Mitochondrial membrane
Mitochondrial envelope
Organelle envelope
Large ribosomal subunit
Ribosome
Ribosomal subunit
Mitochondrion
Inner mitochondrial membrane protein complex
Organelle membrane
Mitochondrial ribosome
Cytoplasm
Intracellular organelle
Intracellular anatomical structure
Organelle
Respiratory chain complex
Mitochondrial respirasome
Protein-containing complex
Signal
Groups at similarity 0.8
FDR
Gene count

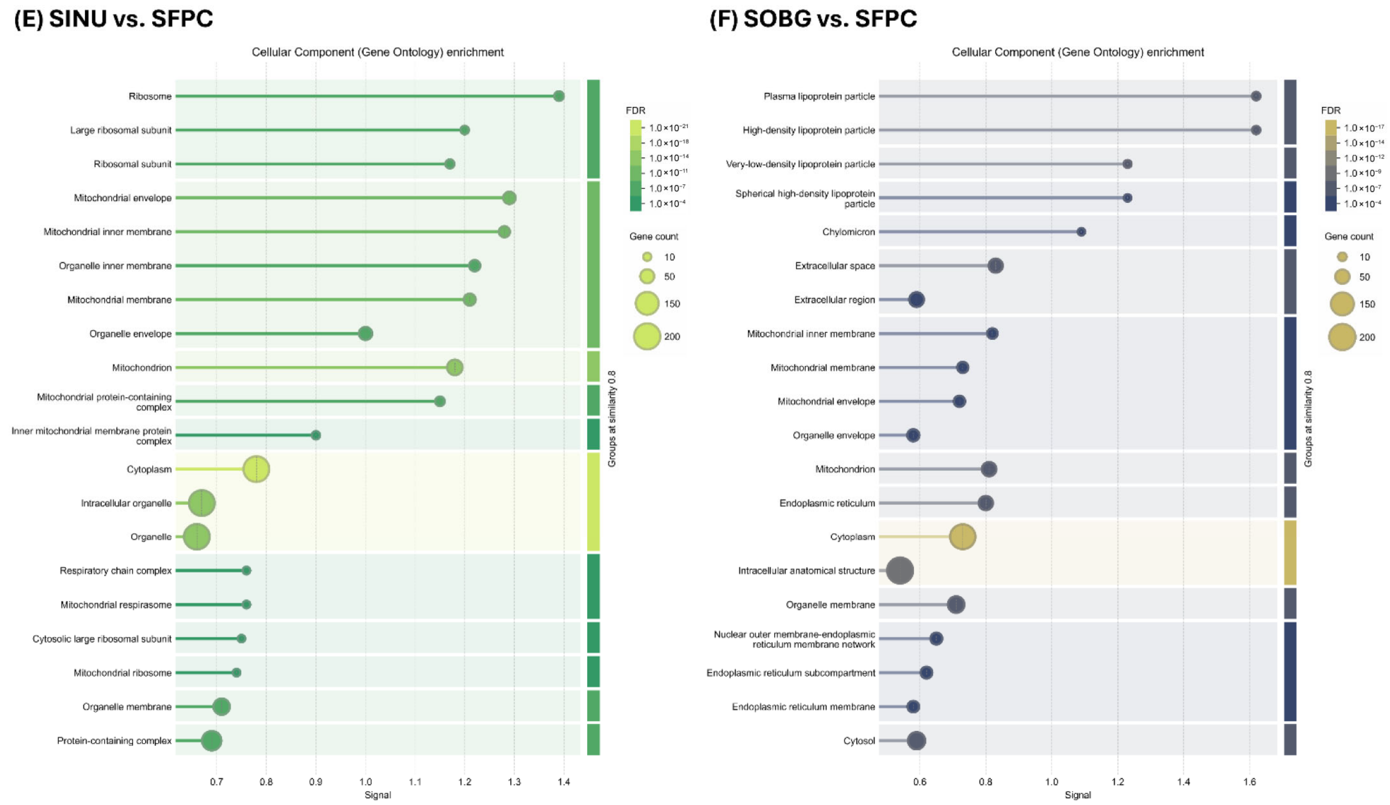

Figure S3. Cellular component (gene ontology) enrichment analysis of total differentially expressed proteins in different group comparisons. (A) NSFPC vs. NSC. (B) SFPC vs. SC. (C) SC vs. NSC. (D) SFPC vs. NSFPC. (E) SINU vs. SFPC. (F) SOBG vs. SFPC. FDR: false discovery rate; NSC: non-shifted chow; NSFPC: non-shifted FPC diet; SC: shifted chow; SFPC: shifted FPC diet; SINU: shifted FPC diet with inulin supplementation; SOBG: shifted FPC diet with oat β-glucan supplementation.
